# The Proteome of Biologically Active Membrane Vesicles from *Piscirickettsia salmonis* LF-89 Type Strain Identifies Plasmid-Encoded Putative Toxins

**DOI:** 10.3389/fcimb.2017.00420

**Published:** 2017-09-28

**Authors:** Cristian Oliver, Mauricio A. Hernández, Julia I. Tandberg, Karla N. Valenzuela, Leidy X. Lagos, Ronie E. Haro, Patricio Sánchez, Pamela A. Ruiz, Constanza Sanhueza-Oyarzún, Marcos A. Cortés, María T. Villar, Antonio Artigues, Hanne C. Winther-Larsen, Ruben Avendaño-Herrera, Alejandro J. Yáñez

**Affiliations:** ^1^Laboratorio de Patología de Organismos Acuáticos y Biotecnología Acuícola, Universidad Andrés Bello, Viña del Mar, Chile; ^2^Facultad de Ciencias, Instituto de Bioquímica y Microbiología, Universidad Austral de Chile, Valdivia, Chile; ^3^Interdisciplinary Center for Aquaculture Research, Concepción, Chile; ^4^Austral-OMICS, Facultad de Ciencias, Universidad Austral de Chile, Valdivia, Chile; ^5^Center of Integrative Microbiology and Evolution, University of Oslo, Oslo, Norway; ^6^Department of Pharmaceutical Biosciences, School of Pharmacy, University of Oslo, Oslo, Norway; ^7^Microbiology and Immunology Department, Dalhousie University, Halifax, NS, Canada; ^8^Department of Biochemistry and Molecular Biology, School of Medicine, University of Kansas Medical Center, Kansas City, KS, United States

**Keywords:** *Piscirickettsia salmonis*, SRS, bacterial toxins, mass spectrometry, MudPIT, zebrafish

## Abstract

*Piscirickettsia salmonis* is the predominant bacterial pathogen affecting the Chilean salmonid industry. This bacterium is the etiological agent of piscirickettsiosis, a significant fish disease. Membrane vesicles (MVs) released by *P. salmonis* deliver several virulence factors to host cells. To improve on existing knowledge for the pathogenicity-associated functions of *P. salmonis* MVs, we studied the proteome of purified MVs from the *P. salmonis* LF-89 type strain using multidimensional protein identification technology. Initially, the cytotoxicity of different MV concentration purified from *P. salmonis* LF-89 was confirmed in an *in vivo* adult zebrafish infection model. The cumulative mortality of zebrafish injected with MVs showed a dose-dependent pattern. Analyses identified 452 proteins of different subcellular origins; most of them were associated with the cytoplasmic compartment and were mainly related to key functions for pathogen survival. Interestingly, previously unidentified putative virulence-related proteins were identified in *P. salmonis* MVs, such as outer membrane porin F and hemolysin. Additionally, five amino acid sequences corresponding to the *Bordetella pertussis* toxin subunit 1 and two amino acid sequences corresponding to the heat-labile enterotoxin alpha chain of *Escherichia coli* were located in the *P. salmonis* MV proteome. Curiously, these putative toxins were located in a plasmid region of *P. salmonis* LF-89. Based on the identified proteins, we propose that the protein composition of *P. salmonis* LF-89 MVs could reflect total protein characteristics of this *P. salmonis* type strain.

## Introduction

Salmonid rickettsial septicemia, also known as piscirickettsiosis, is a multi-systemic infectious disease that produces septicemia in infected salmonids, ultimately affecting the kidney, liver, spleen, intestine, brain, skeletal muscle, ovaries, and gills. This disease causes high mortality rates in Atlantic salmon (*Salmo salar*), coho salmon (*Oncorhynchus kisutch*), and rainbow trout (*Oncorhynchus mykiss*), ultimately translating into significant economic losses for the salmon industry in Chile (Rozas and Enríquez, 2014). Piscirickettsiosis is caused by the Gram-negative, facultative intracellular bacterium *Piscirickettsia salmonis*. This fastidious pathogen was first reported in coho salmon in Chile and has since been found in Canada, Ireland, Norway, and Scotland (Fryer and Hedrick, 2003). Currently, piscirickettsiosis is the most important fish disease affecting marine aquaculture in Chile (Sernapesca, 2015).

Gram-negative bacteria produce membrane vesicles (MVs) during both *in vitro* growth and *in vivo* infection (Lee et al., 2008), including *Escherichia coli, Pseudomonas aeruginosa, Shigella flexneri, Helicobacter pylori* (Hoekstra et al., 1976; Fiocca et al., 1999; Kadurugamuwa and Beveridge, 1999), and the fish pathogens *Francisella noatunensis* (Bakkemo et al., 2011) and *Vibrio anguillarum* (Hong et al., 2009). MVs, small spherical structures that range in size from 10 to 300 nm in diameter, are released from the surface of Gram-negative bacteria. These structures are mainly composed of outer membrane proteins, lipopolysaccharides, phospholipids, and periplasmic proteins and are a reduced composition of inner membrane and cytoplasmic proteins (Deatherage et al., 2009). Interestingly, bacterial MVs can also contain toxins or effector proteins involved in survival and pathogenesis (Bomberger et al., 2009). Indeed, MVs are implicated in the pathogenicity of several bacteria, such as *Acinetobacter baumannii* (Kwon et al., 2009) and *Edwardsiella tarda* (Park et al., 2011). Importantly, MVs have been licensed for use in humans and for example to control outbreaks of disease caused by *Neisseria meningitidis* (Holst et al., 2009, 2013). It was recently reported that *P. salmonis* can produce MVs during normal growth in liquid media and during the infection of CHSE-214 cells. Interestingly, purified MVs are cytotoxic for CHSE-214 cells (Oliver et al., 2016) and zebrafish (*Danio rerio*) (Tandberg et al., 2016). However, despite the increasing research concerning MVs and bacterial pathogenicity, the mechanisms underlying the pathophysiological roles of MVs have not been clearly defined.

Multiple mass spectrometry (MS) methods for the proteomic characterization of bacterial MVs have been reported, including liquid chromatography-MS/MS (Kwon et al., 2009; Pierson et al., 2011; Choi et al., 2014), matrix-assisted laser desorption/ionization, time-of-flight MS (Galka et al., 2008), and multidimensional protein identification technology (MudPIT) (McCaig et al., 2013). Additionally, several proteins involved in virulence were recently pinpointed through the partial proteomic characterization of MVs from the *P. salmonis* LF-89 type strain using liquid chromatography-MS/MS (Oliver et al., 2016; Tandberg et al., 2016). Nevertheless, identification remains pending for the full *P. salmonis*-purified MVs proteome, as well as for toxins or virulence-related proteins that could contribute to pathogenicity. Therefore, the aim of this study was to extensively characterize the proteome of MVs purified from *P. salmonis* LF-89 using highly sensitive MudPIT technology.

## Materials and methods

### Bacterial culture

The *P. salmonis* LF-89 (equivalent to ATCC VR-1361) type strain was grown on AUSTRAL-TSFe agar plates at 18°C for 10 days (Yañez et al., 2013). After this period, bacteria were growth in AUSTRAL- salmonid rickettsial septicemia broth until reaching the logarithmic phase (Yañez et al., 2012). Finally, the culture (4 mL) was inoculated in a minimal liquid medium (400 mL) and incubated at 18°C with agitation (50 rpm) until the early stationary phase (Oliver et al., 2016).

### Isolation and purification of MVs from culture supernatant

MVs were isolated from the culture supernatant following the method described by Oliver et al. (2016). Briefly, *P. salmonis* cells were removed through low-speed centrifugation at 5,000 × g for 10 min at 4°C. The supernatant was sequentially filtered through a 0.45 and 0.22 μm/pore-filter to remove residual cells. Finally, MVs were isolated and concentrated through ultracentrifugation at 125,000 × g for 2 h at 4°C. The pelleted MVs were resuspended in phosphate-buffered saline (PBS) with 0.05% sodium azide. The protein concentration obtained from MVs purification was equivalent to ~166.9 ± 44.5 mg per liter of bacterial culture. The purified MVs were stored at −80°C until use. The purity of MVs after purification was confirmed by transmission electron microscopy.

### Intraperitoneal injection of *P. salmonis*-derived MVs in adult zebrafish

A total of 120 healthy male and female wild-type, strain AB zebrafish (*Danio rerio*; 10–11 months-old) were obtained from the Model Fish Unit at the Norwegian University of Life Science. Fish were acclimatized for 2 weeks at room temperature (20 ± 2°C) prior to the experiment. The fish were fed every morning with brine shrimp (Scanbur AS, Nittedal, Norway) and afternoon with SDS 400 Scientific Fish Food (Scanbur AS). After acclimation, zebrafish were randomly allocated among 6 experimental groups containing 20 fish each. All fish groups were anesthetized by immersion in water containing tricaine methanesulfonate (100 mg/mL; MS-222, Sigma Aldrich St. Louis, MO, USA) buffered with bicarbonate to pH 7–7.5. Then, three experimental groups were intraperitoneally injected (27 G needle) with 20 μL of 10, 20, or 40 μg of *P. salmonis* LF-89 MVs in PBS (Cosma et al., 2006; Brudal et al., 2015). As a positive control, an additional group was intraperitoneally injected with 20 μL of *P. salmonis* LF-89 (equivalent to 10^9^ colony forming units [CFU]/mL). Additionally, a group of 20 fish were injected with PBS as negative control.

After injection, the 6 fish groups (*n* = 20 fish) were separately placed into polycarbonate recovery tanks (6 L; Pentair, Minneapolis, MN, USA), in which 50% of the water was manually changed daily. Tank water was provided by the Model Fish Unit at the Norwegian University of Life Science and was supplemented with Instant Ocean sea salt (0.55 g/L; Spectrum Brands, Blacksburg, VA, USA), sodium bicarbonate (0.053 g/L), and calcium chloride (0.015 g/L). Water parameters (i.e., pH, ${\text{NO}}_{2}^{-}$, ${\text{NO}}_{3}^{2-}$, NH_3_/${\text{NH}}_{4}^{+}$, and hardness) were monitored every third day using commercial TetraTest Kits (Spectrum Brands). The tanks were maintained at 20°C with a 14:10 light:dark cycle. Tank wastewater was decontaminated through chlorination and tested for sterility before disposal.

The fish were closely monitored, and animal health was recorded twice daily. Fish that did not resume normal behavior after injections were removed from the experiment and euthanized with an overdose of tricaine methanesulfonate (250 mg/mL; Sigma Aldrich). These fish included those that were moribund or that clearly showed deviant behaviors/clinical symptoms inconsistent with good animal welfare (e.g., greatly reduced activity levels, environmental responses, and/or appetite). All experimental procedures were approved by The Norwegian Animal Research Authority.

### Sample preparation for proteomics analysis

Purified MVs were incubated in lysis buffer (50 mM Tris–HCl, pH 7.5; 150 mM NaCl; 1% NP-40; 0.5% sodium deoxicolate; and 1% SDS) for 1 h at 4°C. Finally, the solution was sonicated for 10 min at 4°C at a frequency of 20 kHz, lyophilized and stored at −20°C until use. All samples were analyzed by SDS-PAGE.

Lyophilized MVs proteins were resolubilized in 6 M guanidine hydrochloride and 25 mM NH_4_HCO_3_, pH 7.5. Subsequently, proteins were reduced at room temperature for 30 min with 2 mM dithiothreitol and alkylated in the dark at room temperature for 30 min with 10 mM iodoacetamide. The reaction was diluted seven times with 25 mM NH_4_HCO_3_, pH 7.5; 2 μL of 0.1 ng/mL modified trypsin (Promega, Madison, WI, USA) was added, and the reaction was incubated at 37°C for 16 h. The reaction was stopped by adding acetic acid, pH 2.0.

### Identification of MV proteins by MudPIT

All samples were concentrated on a CentriVap Concentrator (Labconco, Kansas City, MO, USA) to a final volume of 20 μL and loaded on a 350 μm ID fused silica 2D high-performance liquid chromatography triphasic peptide trap column packed in-house with 3 cm of a reverse-phase desalting C18 (100 Å, 5 μm Magic C18 particles; Michrom Bioresources, Auburn, CA, USA), 3 cm of a strong cation exchange column (300 Å, 5 μm, PolySULFOETHYL A; PolyLC Inc., Columbia, MD, USA), and, finally, 3 cm of reversed phase resolving C18. The peptide trap was mounted on the loop of a nanoLC (Thermo Finnigan LLC, Waltham, WA, USA). Following a wash with 0.1% formic acid for 30 min at 0.5 μL/min, the efflux of the peptide trap column was directed to a 10 cm resolving reversed-phase column (100 Å, 5 μm Magic C18 particles, Michrom Bioresources), which was mounted on the electrospray stage of a FT ICR mass spectrometer (LTQ FT, Thermo Finnigan LLC). The peptides were separated on-line using 15 salt steps (0, 10, 30, 50, 100, 150, 200, 250, 300, 350, 400, 500, 1,000, 1,500, and 2,000 mM NH_4_CH_3_OO) followed by a 0–90% acetonitrile gradient for 120 min at a flow rate of 350 nL/min. An electrospray voltage of 1.9 kV was used, with the ion transfer temperature set to 250°C. The mass spectrometer was controlled by the Xcalibur software, which continuously performed mass-scan analysis of the FT and, subsequently, of the six most intense ions during MS/MS scans of the ion traps. For this, one repeat scan of the same ion was dynamically excluded, using a 30 s repeat duration and 90 s exclusion duration. Normalized collision energy for the MS/MS was set to 35%. Details of the proteome analysis by MudPIT are available (Supplementary Data 2).

### Data analysis using database search algorithm

All tandem mass spectra MS/MS samples were analyzed using SEQUEST (v1.4.0.288; Thermo Fisher Scientific, San Jose, CA, USA) and X! Tandem (vCYCLONE 2010.12.01.1; The GPM, thegpm.org). SEQUEST searched the National Center for Biotechnology Information (NCBI) *Piscirickettsia salmonis* 12-21-2015.fasta database (10,012 entries) assuming digestion of the enzyme trypsin. X! Tandem searched a subset of the *Piscirickettsia salmonis* NCBI 11-03-2016 database, also assuming trypsin digestion. SEQUEST and X! Tandem were searched with a fragment ion mass tolerance of 0.80 Da and a parent ion tolerance of 50 PPM. Carbamidomethyl-cysteine was a fixed modification in SEQUEST and X! Tandem. In SEQUEST, asparagine and glutamine deamidation and methionine oxidation were variable modifications. In X! Tandem, Glu->pyro-Glu of the N-terminus, ammonia-loss of the n-terminus, gln->pyro-Glu of the N-terminus, asparagine and glutamine deamidation, and methionine oxidation were variable modifications.

### Criteria for protein identification

Scaffold (v.4.5.0; Proteome Software Inc., Portland, OR, USA) was used to validate MS/MS-based peptide and protein identifications. Peptide identifications were accepted if the Peptide Prophet algorithm, with Scaffold delta-mass correction, established a >95.0% probability (Keller et al., 2002). Protein identifications were accepted if presenting a >99.9% probability, as assigned by the Protein Prophet algorithm, and containing at least two identified peptides (Nesvizhskii et al., 2003). Proteins containing similar peptides that could not be differentiated based on MS/MS analysis alone were grouped. Proteins sharing significant peptide evidence were grouped into clusters. Proteins were annotated with NCBI Gene Ontology terms (downloaded 17-03-2016) (Ashburner et al., 2000). Secondary and tertiary structure prediction were make using I-TASSER (https://zhanglab.ccmb.med.umich.edu/I-TASSER/). The crystal structures predictions and alignments were visualized using multiseq extension through VMD 1.9.3 (Visual Molecular Dinamics, Illinois University).

### *In silico* analysis of toxins contained in *P. salmonis* MVs

Amino acid sequences from seven putative proteins annotated as toxins, and separated into two groups, were retrieved from the NCBI database and mapped on the *P. salmonis* LF-89 plasmid 1 (CP011850) using the TBLASTn tool with default parameters. Additionally, five putative pertussis toxin sequences (≈685 amino acids) were aligned using Clustal Omega and phylogenetically analyzed. Phylogenetic calculations and tree building were performed in the CLC Sequence Viewer v7.7 using the UPGMA method and applying a Jukes-Cantor Model. A total of 10,000 bootstrap replicates were performed to evaluate node-support values.

### Statistical analysis

For zebrafish infection, dataset analyses and graphing were completed using Graphpad Prism v7 (GraphPad Software Inc., La Jolla, CA, USA). Mortality curves were used for analyzing the percent mortality, and differences between groups were deemed statistically significant at *p*-value < 0.05, as established using the Gehan-Breslow-Wilcoxon and Log-rank tests.

## Results

### Proteome analysis of MVs derived from *P. salmonis*

MudPIT analysis was performed to identify proteome components in the MVs purified from *P. salmonis* LF-89 (Supplementary Figure 1). A total of 452 unique MV-associated proteins were identified (Supplementary Table 1 and Supplementary Data 1). The 30 most-abundant proteins from the purified MVs are listed in Table 1.

**Table 1:** Most abundant proteins identified from *P. salmonis* LF-89 type strain membrane vesicles.

**Accession (gi)**	**Protein name**	**Molecular weight (kDa)**	**Normalized total spectra^a^**	**Conserved protein domain family**	**Accession function**
838099093	Membrane protein	46	1443	OM channels	cl21487
758740411	Molecular chaperone GroEL	51	113	Chaperonin like	COG0459
873977985	Elongation factor Tu	43	93	P-loop NTPase	cl21455; COG0050; COG2229
873977924	Hypothetical protein PSLF89 280	13	82	Phasin 2	COG5490; cl11491
873979583	Hypothetical protein PSLF89 2434	23	74	OM channels; LomR; LptD	COG1452; cl21487; COG3637
965558488	Superoxide dismutase	18	70	Cu-Zn Superoxide Dismutase	COG2032; cl00891
920728843	Type IV secretion system protein VirB9	39	59	VirB9 CagX TrbG	COG3504; cl11423
920729012	Molecular chaperone DnaK	69	58	DnaK	COG0443
873978943	Cell envelope biogenesis protein OmpA	23	55	OmpA	COG2885
873979466	Disulfide bond formation protein DsbA	30	47	DsbG	COG1651
873976170	Hypothetical protein PSLF89 1p34 (plasmid)	127	44	HepA	COG0553
873980150	Molecular chaperone GroES	11	39	GroES; cpn10	COG0234; cl09113
923110936	Outer membrane porin F	35	36	LomR; OM channels; OmpA	COG3637; cl21487; COG2885
873979967	Peptidylprolyl isomerase	28	34	FkpA	COG0545
738930989	Hypothetical protein	33	33	Pertussis S1	cl03779
920729023	Type IV pilus biogenesis protein PilC	46	30	PulF	COG1459
873979859	Meta-pathway of phenol degradation family protein	20	30	LomR; OM channels	COG3637; cl21487
920731540	D-methionine-binding lipoprotein metQ	31	29	NlpA; Periplasmic binding protein type 2	COG1464; cl21456
314561212	Sequence 14 from patent US 7811583	35	28	AcrA	COG0845
873979142	Conjugal transfer protein TrbI	43	25	VirB10	COG2948
920731472	Membrane protein	17	23	CopD	COG1981; cl21540
738930884	Hypothetical protein	15	22	PAZ	cl00301
873978057	Chemotaxis protein	61	21	Tar	COG0840
873977962	Molecular chaperone DnaJ	40	21	DnaJ	COG0484
965558412	Signal peptidase, peptidase S26 family protein	20	21	TraF; Peptidase S24 S26	COG4959; cl10465
546139961	Major Facilitator Superfamily protein	NR	19	AraJ	COG2814
920728747	Hypothetical protein KW89 30	FNK	19	–	–
873979727	DNA-binding protein	14	18	HimA; HU IHF	COG0776; cl00257
873977977	50S ribosomal protein L1	24	17	RplA; Ribosomal L1	COG0081; cl00322
873978375	Hypothetical protein PSLF89 826	26	16	–	–

a *Relative abundance was calculated from the normalized spectral count for the total of spectra of each identified peptide*.

*NR, not reported; FNK, Function not known*.

**Figure 1:**
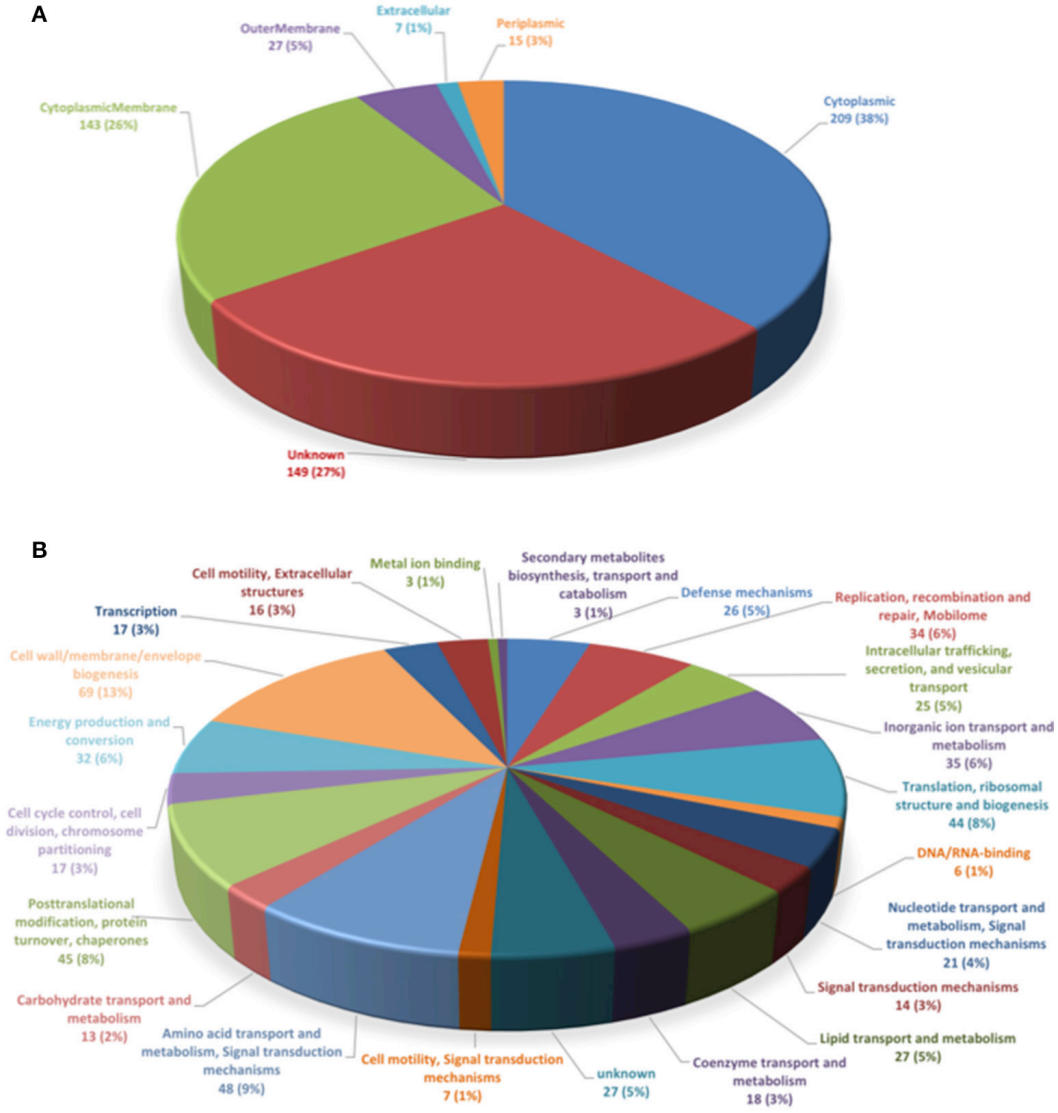
Classification of *P. salmonis* LF-89 membrane vesicles proteins. **(A)** Subcellular locations of membrane vesicle proteins identified by MudPIT. Predicted subcellular locations of the 452 membrane vesicle proteins identified using PSORT3b. **(B)** Functional classification of *P. salmonis* LF-89 membrane vesicles proteins. The 452 proteins identified by MudPIT were sorted according to the indicated clusters of orthologous groups.

To determine the subcellular localization of the identified proteins, the proteins were classified by the subcellular localization prediction tool (PSORTb v.3.0.2). This resulted in the following six groups, which were classified according to protein localization in the bacterium: (1) cytoplasmic proteins, (2) cytoplasmic membrane proteins, (3) periplasmic proteins, (4) outer membrane proteins, (5) extracellular proteins, and (6) proteins of unknown localization or multiple localization sites. Of the 452 proteins identified in MVs, 7 (1.3%) were extracellular, 27 (4.9%) were from the outer membrane, 15 (2.7%) were periplasmic, 143 (26%) were from the inner membrane, 209 (38%) were cytoplasmic, and 149 (27.1%) were from an unknown localization group (Figure 1A). The 5 most-abundant proteins from each subcellular compartment are listed in Table 2. Although a large amount of outer membrane proteins was expected, these results indicate that cytoplasmic proteins are the predominant component in MVs. Furthermore, the high representation of inner membrane and cytoplasmic proteins suggests that *P. salmonis* LF-89 MVs composition is derived from multiple bacterial compartments.

**Table 2:** Five most abundant proteins from each subcellular compartment identified in *P. salmonis* LF-89 type strain membrane vesicles.

**Subcellular compartment**	**Accession (gi)**	**Protein name**	**Molecular weight (kDa)**	**Normalized total spectra^a^**
Outer membrane	KLV35478.1	Membrane protein	46	1,443
	AKP73473.1	Cell envelope biogenesis protein OmpA	23	55
	AKP73996.1	Disulfide bond formation protein DsbA	30	47
	ALB21306.1	Outer membrane porin F	35	36
	WP_032126547.1	Type I secretion protein TolC	51	16
Periplasmic space	AKP73249.2	Superoxide dismutase	18	70
	AKP73751.1	Gamma-glutamyltranspeptidase	63	11
	ALA26413.1	Trypsin family protein	39	8
	ERL62613.1	D-alanyl-D-alanine carboxypeptidase/D-alanyl-D-alanine-endopeptidase	47	3
	ALA24513.1	Tol-Pal system beta propeller repeat protein TolB	48	7
Inner membrane	ALA23777.1	Type IV pilus biogenesis protein PilC	46	30
	ALA26294.1	D-methionine-binding lipoprotein metQ	31	29
	ADS71725.1	Sequence 14 from patent US	35	28
	ALA26226.1	Membrane protein	17	23
	AKP72587.1	Chemotaxis protein	61	21
Cytoplasm	AJO71851.1	Molecular chaperone GroEL	51	113
	AKP72515.1	Elongation factor Tu	43	93
	ALA23766.1	Molecular chaperone DnaK	69	58
	AKP74876.1	Hypothetical protein PSLF89_1p34 (plasmid)	127	44
	AKP74680.1	Molecular chaperone GroES	11	39

a *Relative abundance was calculated from the normalized spectral count for the total of spectra of each identified peptide*.

### Functional classification of identified proteins in *P. salmonis* MVs

To determine the putative functions of the 452 proteins in the *P. salmonis* LF-89 MVs proteome, the proteins were analyzed according to Clusters of Orthologous Groups (COGs) definitions (http://www.ncbi.nlm.nih.gov/COG/). The results showed that the six largest COGs recognized in *P. salmonis* MVs (Figure 1B) were mainly involved in cell wall, membrane, and envelope biogenesis (69 proteins). Furthermore, 48 proteins were involved in the transport and metabolism of amino acids and signal transduction mechanisms; 45 proteins were involved in post-translational modifications, protein turnover, and chaperone activities; 44 were involved in translation, ribosomal structuring, and biogenesis; 35 were involved in inorganic ion transport and metabolism; and 34 proteins were related to the replication, recombination, and repair of the mobilome. Details for the functional classifications of these proteins are shown in Supplementary Table 1. Additionally, some proteins were involved in functions such as defense mechanisms, metal ion binding, and DNA/RNA binding. Interestingly, 25 proteins were identified in relation to intracellular trafficking, secretion, and vesicular transport. Taken together, these results suggest that MVs may exhibit multiple, specific functions inside the host.

### Virulence-associated proteins contained in *P. salmonis* MVs

To gain insight into the virulence potential of *P. salmonis* LF-89 MVs, the 452 proteins identified by MudPIT were subjected to *in silico* analysis using the virulent factor database (Chen et al., 2016), which is designed to predict virulent proteins of pathogenic bacteria. Analysis showed that 64 of the MV proteins (≈14%) (Table 3) had a predicted association with bacterial virulence, including members of the heat-shock families GroEL and GroES, which are strong immunogenic proteins. Additionally, other molecular chaperones, such as GrpE, Hsp33, DnaJ, DnaK, and HtpG, were also identified together with the outer membrane protein OmpA and outer membrane porin OmpF. The latter two are integral outer membrane proteins that are highly immunogenic. Some components of the flagellar structure, such as FlhA, FliF, FliM, FliL, and FliH, and proteins involved in type IV pilus biogenesis, such as PilC, PilT, PilB, PilW, and FimV were found present in *P. salmonis* LF-89 MVs. Furthermore, MVs purified from *P. salmonis* LF-89 also contained siderophores such as SufD, a TonB-dependent siderophore receptor family protein, and other proteins related to iron transport and metabolism. The presence of multiple proteins involved in the secretion of virulence factors supports a role of *P. salmonis* MVs in the pathogenesis of piscirickettsiosis.

**Table 3:** Classification of virulence-related proteins identified from *Piscirickettsia salmonis* LF-89 type strain membrane vesicles.

**Classification**	**Accession (gi)**	**Description**	**Biological function**
Proteases	873980158	ATP-dependent metalloprotease [*P. salmonis* LF-89 = ATCC VR-1361]	ATP-dependent protease
	693576047	Putative endopeptidase Clp ATP-binding chain B [*P. salmonis*]	Clp protease ATP-binding subunit
	873979420	ATP-dependent Clp protease proteolytic subunit [*P. salmonis* LF-89 = ATCC VR-1361]	Clp protease ATP-binding subunit
	873978774	ATP-dependent Clp protease ATP-binding subunit ClpX [*P. salmonis* LF-89 = ATCC VR-1361]	Clp protease ATP-binding subunit
	873977871	Protease [*P. salmonis* LF-89 = ATCC VR-1361]	Protease activity
	920731742	Secreted metalloprotease Mcp02	Elastase LasB
	920731659	Trypsin family protein	Serine protease
Efflux pump	965558557	ABC transporter ATP-binding protein [*P. salmonis* LF-89 = ATCC VR-1361]	ABC transporter ATP-dependent
	546141634	Efflux transporter, RND family, MFP subunit [*P. salmonis* LF-89 = ATCC VR-1361]	RND efflux pump
	546140760	Type I secretion outer membrane, TolC family protein [*P. salmonis* LF-89 = ATCC VR-1361]	TolC family type secretion outer
	873978747	Type I secretion protein TolC [*P. salmonis* LF-89 = ATCC VR-1361]	TolC family type secretion outer
	920729839	Bcr/CflA family drug resistance efflux transporter [*P. salmonis*]	Drug resistance efflux transporter
	546141806	Oligopeptide/dipeptide ABC transporter, ATP-binding, C-terminal domain protein	ABC transporter
	923113856	Multidrug transporter AcrB [*P. salmonis*]	Multidrug transporter
	965557328	Polysaccharide biosynthesis/export family protein	Polysaccharide transmembrane transporter activity
	920731713	MMPL family protein	Cation/multidrug efflux pump
Heat shock proteins	758740409	Molecular chaperone GroEL, partial [*P. salmonis*]	Molecular chaperone
	873977962	Molecular chaperone DnaJ [*P. salmonis* LF-89 = ATCC VR-1361]	Molecular chaperone
	873980150	Molecular chaperone GroES [*P. salmonis* LF-89 = ATCC VR-1361]	Molecular chaperone
	873979102	Molecular chaperone HtpG [*P. salmonis* LF-89 = ATCC VR-1361]	Molecular chaperone
Secretion systems	546141413	Protein-export membrane protein SecD [*P. salmonis* LF-89 = ATCC VR-1361]	Secretion protein
	873979147	Type IV secretion system protein DotC [*P. salmonis* LF-89 = ATCC VR-1361]	Secretion system
	440922721	Dot/Icm type IV secretion system DotA [*P. salmonis*]	Secretion protein
	663090053	Type I secretion outer membrane protein 2 [*P. salmonis*]	Secretion system
	873978747	Type I secretion protein TolC [*P. salmonis* LF-89 = ATCC VR-1361]	Outer membrane protein precursor
	920728841	Type IV secretion system protein VirB4 [*P. salmonis*]	Secretion system
	920730537	Type IV secretion system protein IcmL	Secretion system
	873977714	Preprotein translocase subunit SecA	Secretion protein
	873979155	AAA family ATPase	Dot/Icm type IV secretion system protein IcmB/DotO
	923113388	Type IV secretion system protein IcmO	Secretion protein
	873980314	ATP synthase subunit alpha	ATP synthase in type III secretion system
	873980312	ATP synthase subunit beta	ATP synthase in type III secretion system
Iron metabolism and transport	838099434	Ferric uptake regulator family protein [*P. salmonis* LF-89 = ATCC VR-1361]	Iron regulator
	920730359	Ferric iron reductase FhuF-like transporter family protein [*P. salmonis*]	Transport
	920730832	Iron dicitrate transport regulator FecR [*P. salmonis*]	Iron metabolism
	873979309	Bacterioferritin [*P. salmonis* LF-89 = ATCC VR-1361]	Iron metabolism
	873979364	Iron-sulfur cluster assembly accessory family protein [*P. salmonis* LF-89 = ATCC VR-1361]	Iron metabolism
	873979869	Fe(2+)-trafficking protein [*P. salmonis* LF-89 = ATCC VR-1361]	Iron metabolism
	920730832	Iron dicitrate transport regulator FecR [*P. salmonis*]	Iron metabolism
	965558572	TonB-dependent receptor [*P. salmonis* LF-89 = ATCC VR-1361]	Siderophores metabolism
	920730355	TonB-dependent siderophore receptor family protein [*P. salmonis*]	Siderophores metabolism
	920730153	FeS assembly protein SufD [*P. salmonis*]	Siderophores metabolism
	663090031	Siderophore carboxylate outer membrane receptor [*P. salmonis*]	Siderophores metabolism
Pilus	873978913	Pilus assembly protein PilW [*P. salmonis* LF-89 = ATCC VR-1361]	Secretion
	920729023	Type IV pilus biogenesis protein PilC [*P. salmonis*]	Secretion system
	920729024	Type IV pilus assembly protein PilB [*P. salmonis*]	Secretion system
Flagellar	873979673	Flagellar basal-body rod protein FlgG [*P. salmonis* LF-89 = ATCC VR-1361]	Motion
	920729867	AT hook motif family protein [*P. salmonis*]	Flagelar protein
	920730141	Flagellar biosynthesis protein FlhF [*P. salmonis*]	Motion
	692315022	Flagellar basal body rod protein FlgC [*P. salmonis*]	Motion
	873978423	Flagellar protein export ATPase FliI	ATP synthase in type III secretion system
	873979350	Flagellar biosynthesis protein FlhA	Type III secretion system LcrD homolog protein BcrD
Chemotaxis	546139871	Methyl-accepting chemotaxis (MCP) signaling domain protein	Accessory colonization factor AcfB
	923113559	Chemotaxis methyl-accepting receptor	Toxin co-regulated pilus biosynthesis protein I
Capsule	873978463	Capsule biosynthesis protein	Capsule formation
	546141779	Capsular exopolysaccharide family domain protein	Accessory colonization factor AcfB
Other	920729497	Superoxide dismutase	Superoxide dismutase precursor (Cu-Zn)
	923110936	Outer membrane porin F [*P. salmonis*]	Porin, adhesin
	873980129	D-methionine-binding lipoprotein metQ	Immunogenic lipoprotein A
	920730232	RNA polymerase sigma factor RpoS	Sigma S (sigma 38) factor of RNA polymerase, major sigma factor during stationary phase
	965557432	Penicillin-binding protein activator LpoB [*P. salmonis* LF-89 = ATCC VR-1361]	Antibiotic resistance
	923113583	Penicillin-binding protein 2 [*P. salmonis*]	Antibiotic resistance
	873979221	Gamma-glutamyltranspeptidase	Gamma-glutamyltranspeptidase

### Putative bacterial toxins secreted in *P. salmonis* LF-89 MVs

**Figure 2:**
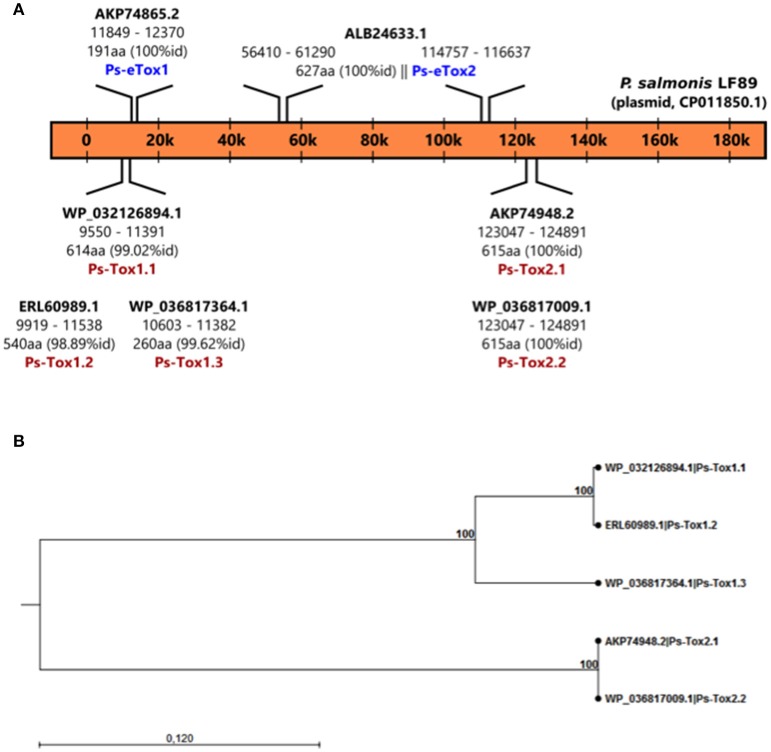
Identification of Ps-Tox genes in the *P. salmonis* LF-89 plasmid. **(A)** Schematic representation of Ps-Tox genes in the *P. salmonis* LF-89 plasmid. Two genomic regions containing three amino acid copies of Ps-Tox 1 (Ps-Tox1.1; Ps-Tox1.2; and Ps-Tox1.3) and two amino acid copies of Ps-Tox 2 (Ps-Tox2.1 and Ps-Tox2.2) were identified after TBLASTn analysis. The identity of each Ps-Tox1 and Ps-Tox2 is indicated in parenthesis. **(B)** Phylogenetic relationship between five Ps-Tox copies. A phylogenetic tree was constructed using the neighbor joining method with 1,000 bootstrap replicates according to the alignment of the Ps-tox amino acid sequence. Bootstrap support values are indicated at the nodes.

A total of seven putative toxin sequences (Table 4) were detected in the proteome of MVs purified from *P. salmonis* LF-89 through an NCBI conservative domain database search (http://www.ncbi.nlm.nih.gov/Structure/cdd/cdd.shtml). The identified toxin-related peptides are listed in Supplementary Table 2. Five amino acids sequences corresponding to *Bordetella pertussis* toxin subunit 1 were identified in the *P. salmonis* LF-89 plasmid pPSLF89-1 (accession number CP011850.1) (Figure 2A). Interestingly, three of these sequences (i.e., WP_032126894.1, ERL60989.1, and WP_036817364.1) corresponded to Ps-Tox1 and were located in the same plasmid region (between ≈9,500 and 11,600 base pairs). TBLASTn analyses of these sequences showed high identity percentages (>95%) with the *P. salmonis* LF-89 plasmid sequence. Similarly, two identical sequences (accession number AKP74948.2 and WP_036817009.1) corresponding to Ps-Tox2 were located in the plasmid region between 123,047 and 124,891 base pairs, with 100% identity. Additionally, multiple alignments of amino acid sequences showed that Ps-Tox1.1, Ps-Tox1.2, and Ps-Tox1.3 were identical to the *B. pertussis* toxin subunit 1 (accession number AMT50644.1), evidencing 7.5, 8.9, and 11.6% identities, respectively (Supplementary Figure 2 and Supplementary Data 3). In the case of Ps-Tox2.1 and Ps-Tox2.2, the identity percentage was 8.6%. Despite the low identities of these putative toxins, most changes in the amino acids sequences were conservative modifications. Subsequently, a phylogenetic tree was constructed according to amino acid sequence alignment of the five putative toxins corresponding to Ps-Tox (Figure 2B). As could be expected from the previously obtained data, these putative toxins were classified into two clusters, namely Ps-Tox1, containing Ps-Tox1.1, Ps-Tox1.2, and Ps-Tox1.3; and Ps-Tox2, conformed by Ps-Tox2.1 and Ps-Tox2.2.

**Table 4:** Toxins identified by MudPIT analysis from *Piscirickettsia salmonis* LF-89 type strain membrane vesicles.

**Accession number^a^**	**Previous annotation**	**New annotation**	***E*-value^b^**	**Bit Score^c^**	**Accession number^d^**	**Short name**	**Definition**
ERL60989	Hypothetical protein K661_02693, partial	Ps-Tox1	3.10E-04	41,286	cl03779	Pertussis_S1 superfamily	Pertussis toxin, subunit 1 *Bordetella pertussis*
WP_032126894	Hypothetical protein	Ps-Tox1	2.40E-04	416,712	cl03779	Pertussis_S1 superfamily	Pertussis toxin, subunit 1 *B. pertussis*
WP_036817364	Hypothetical protein	Ps-Tox1	1.90E-03	37,434	cl03779	Pertussis_S1 superfamily	Pertussis toxin, subunit 1 *B. pertussis*
WP_036817009	Hypothetical protein	Ps-Tox2	1.00E-04	428,268	cl03779	Pertussis_S1 superfamily	Pertussis toxin, subunit 1 *B. pertussis*
AKP74948	Hypothetical protein PSLF89_1p159 (plasmid)	Ps-Tox2	1.15E-04	428,268	cl03779	Pertussis_S1 superfamily	Pertussis toxin, subunit 1 *B. pertussis*
AKP74865.2	Hypothetical protein KU39_3p171 (plasmid)	Ps-eTox1	3.18E-04	413,979	cl03186	Enterotoxin_a superfamily	Heat-labile enterotoxin alpha chain *E. coli*
ALB24633.1	Hypothetical protein PSLF89_1p15 (plasmid)	Ps-eTox2	3.50E-03	356,199	cl03186	Enterotoxin_a superfamily	Heat-labile enterotoxin alpha chain *E. coli*

a *Gene bank accession number from NCBI*.

b *Statistical significance of the hit as hit likelihood was found by chance*.

c *Raw alignment score*.

d *Gene ontology accession number; conserved domains from NCBI*.

On the other hand, another two amino acid sequences corresponding to the heat-labile enterotoxin alpha chain of *E. coli* were also identified in the *P. salmonis* LF-89 plasmid. One sequence (100% identity) for Ps-eTox1 (accession number AKP74865.2) was located in the plasmid region between ≈11,850 and 12,370 base pairs, while the second amino acid sequence (100% identity) was for Ps-eTox2 (accession number ALB24633.1) and matched two different plasmid regions (≈56,400 and 61,300 base pairs, and ≈114,750 and 116,640 base pairs). Multiple alignments of these amino acid sequences showed that Ps-eTox1 and Ps-eTox2 were identical to the enterotoxin alpha from *E. coli* (13.7 and 16.6%, respectively) (Supplementary Figure 3).

Interestingly, the analysis of the secondary and tertiary structure of Ps-Tox and Ps-eTox generated by i-tasser revealed a high structural similarity with CARDS toxin, which is present in *Mycoplasma pneumonia*, and also common with the adhesion domain of *B. pertussis* toxin and heat-labile enterotoxin alpha chain of *E. coli* (Supplementary Figure 4).

### Effect of MVs isolated from *P. salmonis* in adult zebrafish

**Figure 3:**
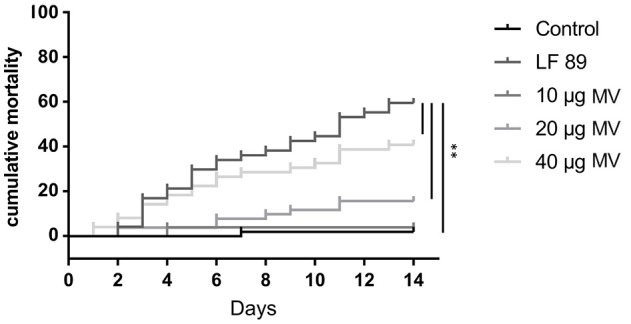
Cumulative mortality (%) of adult zebrafish challenged with membrane vesicles (MVs) isolated from *Piscirickettsia salmonis* LF-89 type strain. Adult zebrafish were injected with 10, 20, and 40 μg of MVs isolated from *P*. *salmonis*. PBS and live *P. salmonis* LF-89 were used as controls (*n* = 20). Asterisks indicate statistical significance (*P* < 0.05).

To evaluate the toxicity of MVs isolated from the pathogenic *P. salmonis* LF89 strain, different MV concentrations were injected into an adult zebrafish infection model. The cumulative mortality of zebrafish injected with MVs showed a dose-dependent pattern (Figure 3). Fish injected with 10 μg of MVs registered < 5% mortality 14 days post-injection. In contrast, fish injected with 40 μg of MVs showed a rapid onset of mortalities (≈20%) just 3 days post-injection. After 14 days, the 40 μg MV group registered ≈45% mortality, which was higher than the final 5 and 15% in the 10 and 20 μg MV groups, respectively. Interestingly, zebrafish challenged with live *P. salmonis* (10^9^ CFU/mL, positive control) had a cumulative mortality of ≈30% 7 days post-injection, a rate similar to that for the 40 μg MV group. Nevertheless, the mortality of the positive control group reached ≈60% by the end of the challenge period. These findings support that MVs isolated from *P. salmonis* are cytotoxic for zebrafish.

## Discussion

Membrane vesicles are produced by several Gram-negative bacteria, and the pathogenic role of MVs during bacterial infection has been extensively reported (Lim and Yoon, 2015). The main focus of this study was to expand on the knowledge available for proteins contained in *P. salmonis* MVs. In the present work, a total of 452 proteins were identified using MudPIT analysis, 28 of which were within the outer membrane, as indicated by the PSORTb algorithm. Interestingly, most proteins (209) in *P. salmonis* LF-89 MVs corresponded to the cytoplasmic compartment, and 143 proteins were associated with the inner membrane. Finally, the locations of 149 proteins could not be identified. Despite some reports suggesting that Gram-negative bacterial MV proteomes consist mainly of outer membrane and periplasmic proteins (Horstman and Kuehn, 2000; Deatherage et al., 2009), other proteomic approaches indicate that MVs contain proteins with different cellular origins, including the cytoplasmic, inner membrane, outer membrane, and periplasmic proteins (Lee et al., 2007; Galka et al., 2008). Other studies still report that MVs contain mostly cytoplasmic and periplasmic proteins (Choi et al., 2011; Bai et al., 2014), such as specifically observed in the *Aggregatibacter actinomycetemcomitans* MV proteome (Kieselbach et al., 2015). A possible explanation for this phenomenon may be related to a physiological role of MVs, which have been found to help bacteria expel useless and harmful waste that has accumulated inside bacterial cells via MVs (McBroom and Kuehn, 2007). However, further studies are needed to elucidate this hypothesis and the production of MVs by *P. salmonis*. On the other hand, the presence of cytoplasmic proteins in the MV proteome may be due to moonlighting abilities, which is the capacity to perform additional biological activities distinct from those they normally occupy (Mani et al., 2014). These multitasking bacterial proteins include metabolic proteins/enzymes and molecular chaperones, which could to play a role in bacterial interaction with host cells by serving as adhesins and invasins (Henderson and Martin, 2011; Wang et al., 2014). Thus, this possibility needs to be assessed through further studies on *P. salmonis* MV cytoplasmic proteins and respective potential moonlighting functions. Nevertheless, the present findings support that the *P. salmonis* LF-89 MV proteome includes proteins from different subcellular origins.

In relation to differential origins, the identified *P. salmonis* LF-89 MV proteins presented varied functions. Thus, a total of 69 proteins were classified through COG definitions with functions in cell wall, membrane, and envelope biogenesis, including VirB9, required for type IV secretion (Jakubowski et al., 2003), which has been shown to induce humoral and cellular immunity in *A*. *marginale* (Zhao et al., 2016). Likewise, the inner membrane-associated ATPase VirB4, essential for pilus biogenesis and protein transport in type IV secretion systems was also identified (Peña et al., 2012). In turn, 48 proteins were related to amino acids transport and metabolism, as well as to signal transduction mechanisms. Other proteins were associated with functions of post-translational modification, protein turnover, and chaperone activity (45 proteins), including GroEL, GroES, DnaJ, and HtpG, homolog of the ubiquitous HSP90 family of proteins; translation, ribosomal structure, and biogenesis (44 proteins); inorganic ion transport and metabolism (35 proteins); and the replication, recombination, and repair of the mobilome (34 proteins). Additionally, other proteins were involved in defense mechanisms, metal ion binding, and DNA/RNA binding. Overall, the high amount of *P. salmonis* MV proteins involved in key functions for pathogen survival is in accordance with findings from previously reported MV proteomes for several Gram-negative bacteria, such as *Pseudomonas syringae* (Kulkarni et al., 2014) and *A. actinomycetemcomitans* (Kieselbach et al., 2015).

From a functional point of view, many vesicle-associated proteins are virulence factors, playing diverse bacterial roles in pathogenicity such as invasion, adherence, antibiotic resistance, damage to host cells, modulation of the host immune response, biofilm formation, and promotion of virulence. Thus, several antibiotic resistance-related proteins have been identified in the *P. salmonis* MVs proteome including the transporter AcrB, TolC, and the MFP subunit, members of the RND-type multidrugs efflux pumps, which have been previously described in *P. salmonis* (Sandoval et al., 2016). Furthermore, the Bcr/CflA family drug resistance efflux transporter, described as resistance to bicyclomycin in *E. coli* (Bentley et al., 1993), and chloramphenicol and florfenicol in *Salmonella typhimurium* (Braibant et al., 2005) were also identified. Additionally, several proteins involved in iron metabolism and uptake (FhuF-like transporter, the regulator FecR, and Bacterioferritin), and siderophores metabolism (TonB-dependent siderophore receptor and FeS assembly protein SufD) were identified. These proteins are highly important for intracellular bacterial pathogens, which use multiple strategies to obtain nutritional iron from the intracellular environment in order to use this element for its replication, in the same way as it does *P. salmonis* (Pulgar et al., 2015; Almarza et al., 2016). Although, the flagellar basal-body rod protein Flagellin G (FlgG) and the chaperone GroEL are present in *P. salmonis* MVs and they were chosen in early vaccine studies (Wilhelm et al., 2006), the field results suggest that these two proteins are not suitable as a vaccine candidates for *P. salmonis*. It is possible that some combination of these and other immunogenic and/or virulence-associated antigens may be needed as has been reported for the fish pathogen *Flavobacterium psychrophilum* (Plant et al., 2011).

Interestingly, our study identified the outer membrane proteins OmpA, that has been involved in adhesion, invasion and replication of several bacterial pathogens; and OmpF, with porin activity forming small water-filled channels (Buehler et al., 1991; Cowan et al., 1992). These highly immunogenic proteins are found across genera in Gram-negative bacteria such as *Yersinia enterocolitica* (Gu et al., 2012), *Salmonella enterica* (Toobak et al., 2013), and *Coxiella burnetii* (Martinez et al., 2014), and several successful bacterial OMP-based vaccines have used OmpA and OmpF in its formulation (Camacho et al., 2013; Liu et al., 2016). Thus, *P. salmonis* MVs containing OmpA and OmpF proteins could serve as protective antigens and should be further assessed as potential vaccine candidates against piscirickettsiosis.

Furthermore and importantly, this is the first study demonstrating that the *B. pertussis* toxin subunit 1 and heat-labile enterotoxin alpha chain of *E. coli* are proteins carried by *P. salmonis* LF-89 MVs. It has been widely demonstrated that important bacterial toxins are secreted via bacterial MVs, including heat-labile enterotoxin from *E. coli* (Horstman and Kuehn, 2000), the anthrax toxin from *Bacillus anthracis* (Rivera et al., 2010), the cholera toxin from *Vibrio cholerae* (Chatterjee and Chaudhuri, 2011), listeriolysin O (*Listeria monocytogenes*), and alpha-hemolysin from *Staphylococcus aureus* (Lee et al., 2013). More specifically, *B. pertussis* toxin subunit 1 (28 kDa) is an important virulence factor that exercises NAD-dependent ADP-ribosyltransferase activity, which plays a crucial role in *B. pertussis* pathogenesis by causing the suppression/modulation of the host immune and inflammatory responses (Higgs et al., 2012; Melvin et al., 2014). Indeed, ADP-ribosylation of target substrates in eukaryotic cells is a common action mechanism of many bacterial protein toxins, including the cholera toxin from *V. cholerae* (Chatterjee and Chaudhuri, 2011) and exotoxin A from *P. aeruginosa* (Allured et al., 1986). In turn, expression of the heat-labile enterotoxin by enterotoxigenic *E. coli* promotes bacterial adherence to intestinal epithelial cells (Johnson et al., 2009), causing diarrhea in infected subjects (Nataro, 2005). This action is mediated by an ADP-ribosylation activity of the A subunit of heat-labile enterotoxin. Additionally, further reports support that enterotoxigenic *E. coli* secretes physiologically active heat-labile enterotoxin via MVs (Horstman and Kuehn, 2000). Similar to *P. salmonis*, it was recently found that *B. pertussis* can survive and replicate inside human macrophages. Furthermore, the bactericidal and inflammatory response of infected macrophages is progressively downregulated, and the pertussis toxin is involved in manipulating the host-cell response (Valdez et al., 2016). Likewise, once *P. salmonis* is inside the host cell, can modulate the expression of several pro-inflammatory cytokines (Tacchi et al., 2011; Salazar et al., 2016). Furthermore, IL-10 is upregulated in the RTS-11 monocyte/macrophage cell line during *P. salmonis* infection, thus promoting the bacterial survival inside the cell through macrophage inactivation (Álvarez et al., 2016). Likewise, Tandberg et al. (2016) demonstrated an upregulation of several pro-inflammatory genes in the spleen and kidney of adult zebrafish after immunization with MVs from *P. salmonis*. Additionally, it has been revealed that *tnf-a, il-1b, il-6* and *il-10* display significant differences in MV-immunized fish (Tandberg et al., 2017). The modulation of these genes might therefore indicate the functionality of *B. pertussis* toxin subunit 1 and *E. coli* heat-labile enterotoxin alpha in the modulation of the host immune response and in the pathogenicity of *P. salmonis*. However, the presence of these putative toxins in *P. salmonis* MVs, the toxicity induced by these toxins, toxin regulations, and the modulation of the host immune and inflammatory responses by these putative toxins should be explored in future studies.

Our group previously reported the production of MVs by the fish pathogen *P. salmonis* through microscopic characterization and liquid chromatography-MS/MS, the first proteomic approach in this bacterium (Oliver et al., 2016). In the present study, the cytotoxicity of MVs purified from the *P. salmonis* LF-89 type strain was confirmed in an *in vivo* model. Specifically, the cumulative mortality of adult zebrafish was ≈40% 14 days after MV injection (40 μg). This finding was similar to Tandberg et al. (2016), who reported a mortality of ≈50% in zebrafish injected with the same quantity of MVs purified from *P. salmonis* isolates. Additionally, the presently purified MVs induced dose-dependent mortality rates in fish. However, the inflammatory response, other immune issues, and the putative protection induced by the MVs, a point imperative to the possible vaccine application of MVs against piscirickettsiosis, were not evaluated in this study and should be considered in future investigations.

On the other hand, MV-based vaccines have successfully been used for epidemic control against serogroup B meningococcal disease (Holst et al., 2013). MVs used in vaccination of fish have also been reported to give good protection against several fish pathogens (Lagos et al., 2017; Tandberg et al., 2017), inducing up-regulation of immune-related genes, showing MVs as potential activator of the host's immune system. However, whether this activation is mediated by i.e., toll-like receptors (TLRs) or not, is still not known. Thus, considering the composition of MVs, which contain several molecules and proteins identified as pathogen associated molecular pattern (PAMPS) including LPS, carbohydrates, HSPs, and nuclei sequence motifs suggest the participation of TLRs as a bridge between innate and adaptive immunity, making *P*. *salmonis* MVs interesting as a vaccine candidate. Interestingly, it has been described different effects on mortality induced by MVs purified from three different *P. salmonis* strains, been LF-89 MVs the most toxic (Tandberg et al., 2016). However, whether the difference in mortality are caused by differences in the LPS, it is unknown. Thus, there is still a lack of knowledge regarding the immunogenic effect of LPS from fish pathogens, and studies of *P*. *salmonis* derived LPS would be interesting to follow up in future studies Recent studies have shown that LPS is one of the most abundant components of OMVs, being able to exceed the total protein content of vesicles by ratios as high as 10:1 (Ellis and Kuehn, 2010). Given the high LPS content, all investigations into immune responses to OMVs must define the contribution of LPS to the host response. MVs, as LPS delivery vehicles, have the capacity to enhance either bacterial clearance or cause host tissue damage by activating an inflammatory response. Recent studies have identified MVs as the vehicle that mediates the cytosolic localization of LPS during extra cellular Gram-negative bacterial infections, demonstrating a necessary role for MVs for intracellular LPS release during bacterial infections (Vanaja et al., 2016). To date, no studies have demonstrated LPS purified from either *P. salmonis* or MVs directly impacting the host responses. However, the present study identified several proteins in MVs, including toxins, which would be able to stimulate the fish immune system. However, the importance of each of these components in virulence and pathogenesis of *P. salmonis* it is until now, unexplored.

Collectively, these results suggest that MV secretion might have an association with *P. salmonis* virulence. Although not specifically tested herein, we speculate that MVs secretion might contribute to the transport and dissemination of key virulence factors and putative bacterial toxins to host cells during bacterial infection.

In conclusion, the present study identified 452 proteins in *P. salmonis* MVs, which, to our knowledge, is the most comprehensive report on a bacterial MV proteome. Notably, a relatively large number of cytoplasmic proteins were found in the vesicles. Taken together, the present results support that the *P. salmonis* MVs purified from the LF-89 type strain contain numerous virulence factors that can stimulate the host immune system, as well as some proteins involved in antibiotic resistance, invasion into host cells, and, interestingly, intracellular trafficking. Two putative toxins were also identified in the MVs, which might be involved in *P. salmonis* cytotoxicity, as previously reported by our research group. Overall, the currently presented results suggest that the protein composition of the MVs in *P. salmonis* LF-89 may reflect the characteristics of the total *P. salmonis* proteome. This valuable information provides a basis for future studies toward elucidating key pathogenic roles of *P. salmonis* MVs. Moreover, this study should contribute to the development of vaccines or vaccine adjuvants against this fastidious fish pathogen.

## Author contributions

CO planned and performed most of the experiments and participated in the writing of the manuscript. MH, JT, KV, LL, RH, and PS planned and performed some of the experiments. CS, MC, and PR performed some of the experiments and participated in the writing of the manuscript. MV, AA, HW, RA, and AY planned some of the experiments and participated in writing of the manuscript. All authors have approved the final article.

### Conflict of interest statement

The authors declare that the research was conducted in the absence of any commercial or financial relationships that could be construed as a potential conflict of interest.
