# Supplementary material for: The Proteome of Biologically Active Membrane Vesicles from Piscirickettsia salmonis LF-89 Type Strain Identifies Plasmid-Encoded Putative Toxins
Source: Front Cell Infect Microbiol. 2017 Sep 28;7:420. doi: 10.3389/fcimb.2017.00420 (PMC5625009; doi:10.3389/fcimb.2017.00420)
Supplement: Supplementary file 1 [file Presentation1.pdf]

Supplementary Material

Biologically Active Membrane Vesicles Mediate Transport of Plasmid-Encoded Toxins from *Piscirickettsia salmonis* LF-89 Type Strain

Cristian Oliver, Mauricio Hernández, Julia I. Tandberg, Karla Valenzuela, Leidy X. Lagos, Ronie E. Haro, Patricio Sánchez, Pamela Ruiz, Constanza Sanhueza-Oyarzún, Marcos Cortés, María T. Villar, Antonio Artigues, Hanne C. Winther-Larsen, Ruben Avendaño-Herrera*, Alejandro J. Yáñez*

*** Correspondence:** Ruben Avendaño-Herrera: reavendano@yahoo.com, ravendano@unab.cl
Alejandro Yañez: ayanez@uach.cl

1 Supplementary Data

Supplementary Data 1. Peptide report for scaffold MudPIT annotations. Complementary data to Supplementary Table 1.

Supplementary Data 2. Batch CD depicting step-by-step methodology for protein analysis.

Supplementary Data 3. Subcellular localization of proteins identified through GO annotation. Complementary data to Figure 2 in main manuscript.

2 Supplementary Figures and Tables

2.1 Supplementary Tables

Supplementary Table 1. Protein report for scaffold MudPIT annotations.

Supplementary Table 2. Peptides that match the toxins sequence of *P. salmonis* OMVs identified by MudPIT.

2.2 Supplementary Figures

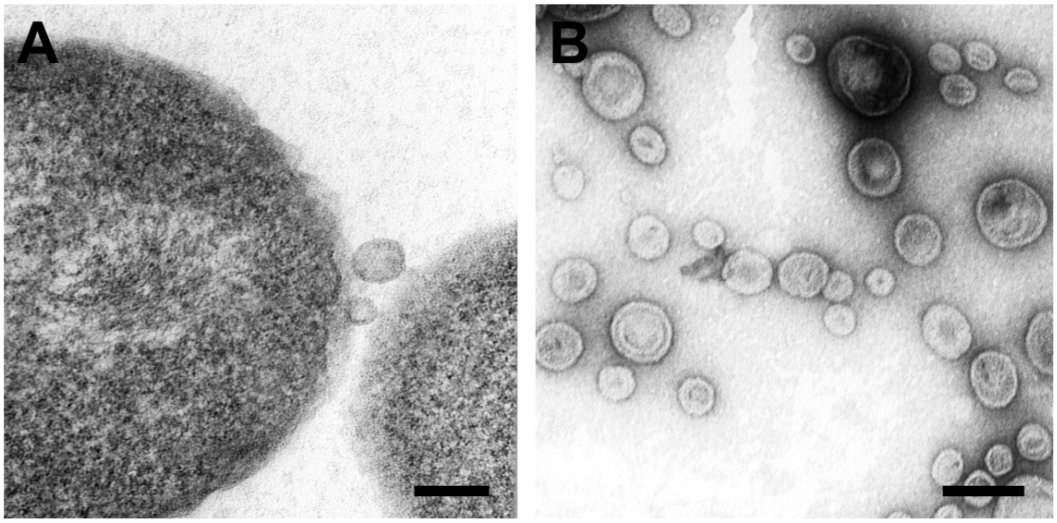

Supplementary Figure 1. MVs purified from *P. salmonis* LF-89 type strain. **(A)** TEM image of MVs released by *P. salmonis* grown in liquid medium. **(B)** Negative stain of MVs purified from supernatant of *P. salmonis* culture. Bars: 100 nm.

frontiers

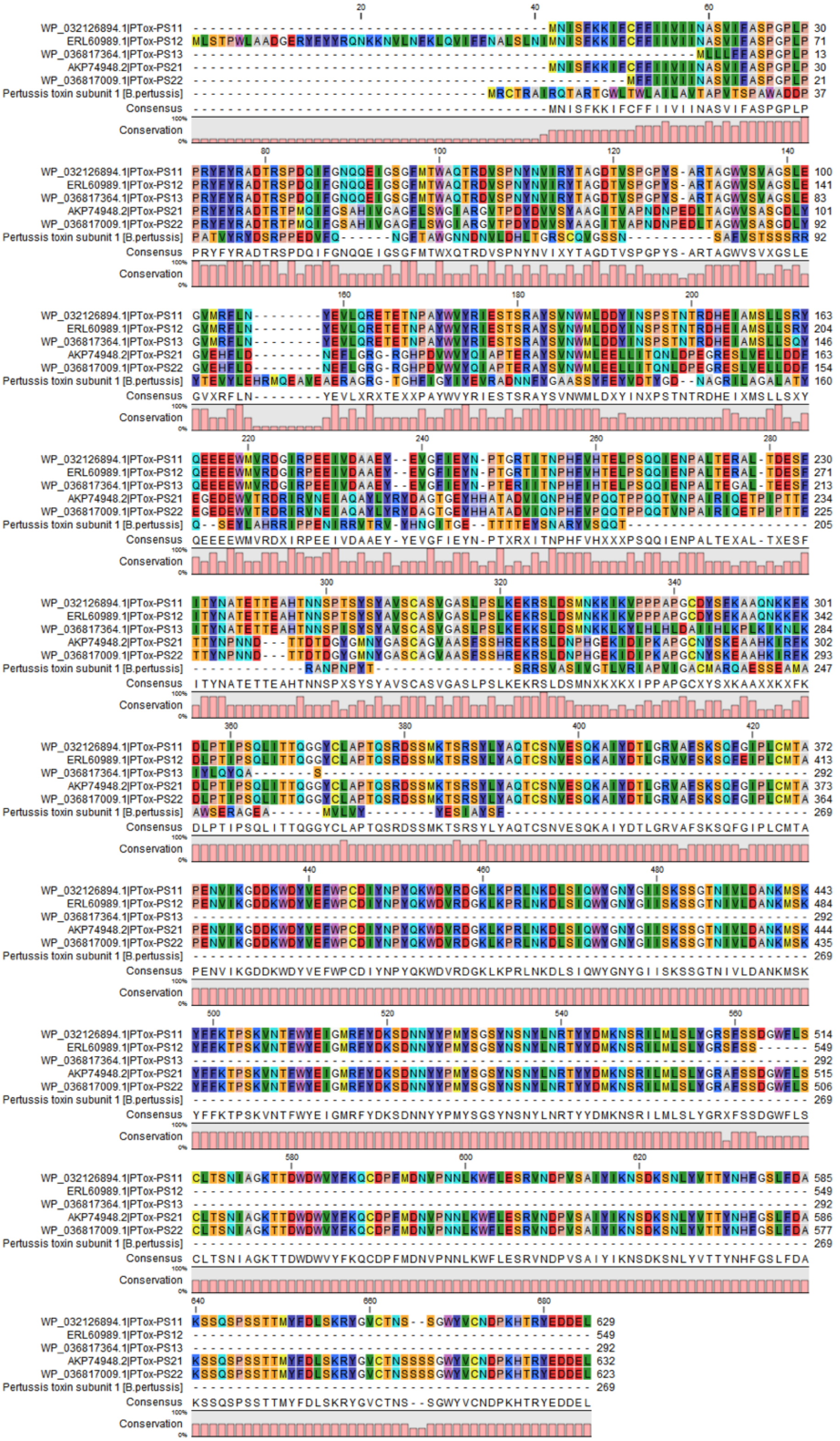
WP_032126894.1|PTox-PS11
ERL60989.1|PTox-PS12
WP_036817364.1|PTox-PS13
AKP74948.2|PTox-PS21
WP_036817009.1|PTox-PS22
Pertussis toxin subunit 1 [B.pertussis]
Consensus
Conservation

Supplementary Figure 2. Multiple sequence alignment analysis of *P. salmonis* Ps-Tox. Protein sequence comparison for each Ps-Tox type identified by MudPIT analysis was made using CLC Genomics Workbench software version 7.7.

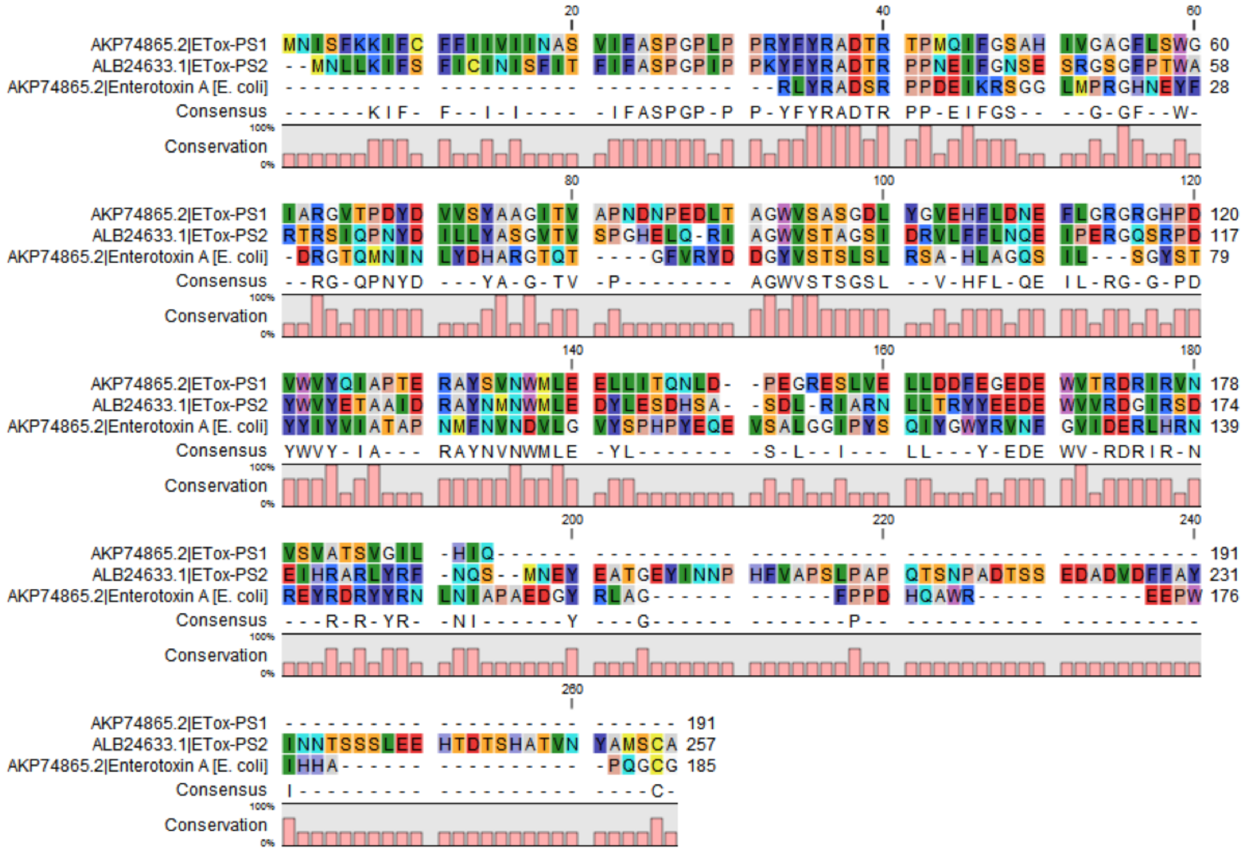

Supplementary Figure 3. Multiple sequence alignment analysis of *P. salmonis* Ps-ETox-like. Protein sequence comparison for each Ps-ETox-like type identified by MudPIT analysis was made using CLC Genomics Workbench software version 7.7.

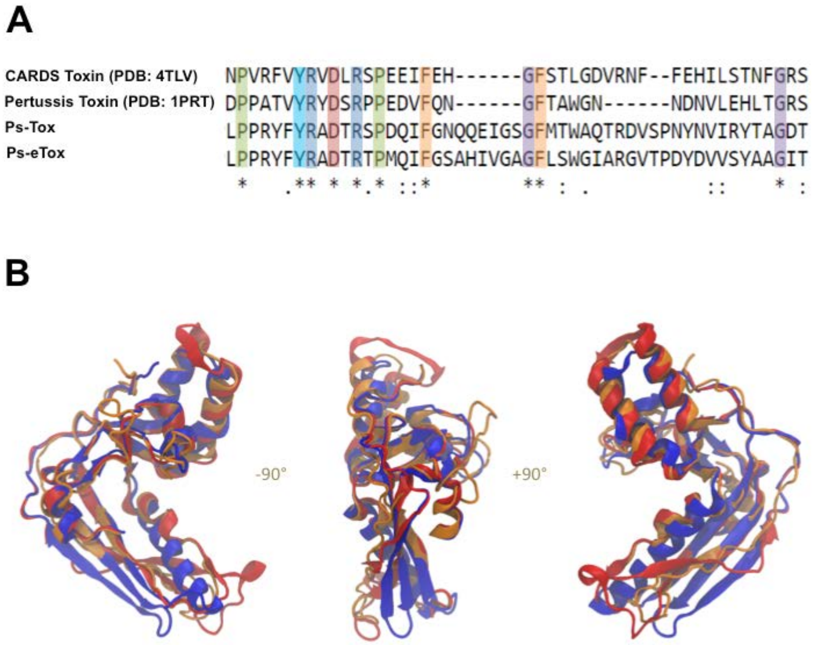

Suppl. Fig. 4. Structural analysis of toxins identified in *P. salmonis* MVs. **(A)** Multiple alignment of a conserved domain sequences of the analyzed proteins using as template CARDS Toxin domain (*Mycoplasma pneumoniae*). **(B)** Structural alignment between CARDS toxin domain (PDB: 4TLV, red), alpha chain pertussis toxin (PDB: 1PRT, blue), and Ps-Tox (orange) in different views.
