# Supplementary material for: The Proteome of Biologically Active Membrane Vesicles from Piscirickettsia salmonis LF-89 Type Strain Identifies Plasmid-Encoded Putative Toxins
Source: Front Cell Infect Microbiol. 2017 Sep 28;7:420. doi: 10.3389/fcimb.2017.00420 (PMC5625009; doi:10.3389/fcimb.2017.00420)
Supplement: Supplementary file 3 [file Table2.docx]

**Supplementary Table 2.** Peptides that match the toxins sequence of *P. salmonis* OMVs identified by MudPIT.

| **Toxin**  **Peptide sequence** | **XCorr^a^** | **Charge^b^** | **m/z**  **(Da)^c^** | **RT**  **(min)^d^** |
| --- | --- | --- | --- | --- |
| **Ps-Tox1** |  |  |  |  |
| WDYVEFWPcDIYNPYQK | 3.04 | 2 | 2323.1187 | 51.73 |
| SYLYAQTcSNVESQK | 3.03 | 2 | 1777.85198 | 28.18 |
| **Ps-Tox1** |  |  |  |  |
| WDYVEFWPcDIYNPYQK | 3.04 | 2 | 2323.1187 | 51.73 |
| SYLYAQTcSNVESQK | 3.03 | 2 | 1777.85198 | 28.18 |
| **Ps-Tox1** |  |  |  |  |
| ESLVELLDDFEGEDEWVTR | 5.00 | 2 | 2281.14409 | 73.95 |
| SYLYAQTCSNVESQK | 4.73 | 2 | 1777.88396 | 27.67 |
| YDAGTGEYHHATADVIQNPHFVPQQTPPQQTVNPAIR | 3.51 | 5 | 4100.13135 | 36.62 |
| WDYVEFWPCDIYNPYQK | 3.04 | 2 | 2323.11870 | 51.73 |
| FKDLPTIPSQLITTQGGYCLAPTQSR | 2.84 | 3 | 2893.59464 | 39.61 |
| SSGTNIVLDANK | 2.20 | 1 | 1218.68420 | 30.76 |
| FKDLPTIPSQLITTQGGYCLAPTQSR | 2.51 | 3 | 2894.54148 | 48.20 |
| **Ps-Tox2** |  |  |  |  |
| ESLVELLDDFEGEDEWVTR | 5.00 | 2 | 2281.144091 | 73.95 |
| SYLYAQTcSNVESQK | 4.73 | 2 | 1777.883959 | 27.67 |
| YDAGTGEYHHATADVIqNPHFVPQQTPP-QqTVNPAIR | 3.51 | 5 | 4100.131355 | 36.62 |
| YDAGTGEYHHATADVIQNPHFVPqqTPP-qQTVNPAIR | 3.23 | 4 | 4101.042135 | 36.40 |
| WDYVEFWPcDIYNPYQK | 3.04 | 2 | 2323.1187 | 51.73 |
| SSQSPSSTTmYFDLSK | 2.72 | 2 | 1781.804003 | 22.97 |
| SSGTNIVLDANK | 2.20 | 1 | 1218.684204 | 30.76 |
| DLPTIPSQLITTqGGYcLAPTQSR | 2.01 | 3 | 2618.351597 | 42.77 |
| VNEIAQAYLYR | 1.80 | 2 | 1339.713549 | 24.55 |
| VNDPVSAIYIK | 1.65 | 2 | 1218.681688 | 25.71 |
| FKDLPTIPSqLITTQGGYcLAPTQSR | 2.84 | 3 | 2893.594639 | 39.61 |
| SQFGIPLcMTAPENVIK | 1.74 | 2 | 1905.038011 | 45.91 |
| FKDLPTIPSqLITTqGGYcLAPTQSR | 2.51 | 3 | 2894.541477 | 48.20 |
| QcDPFMDNVPNNLK | 1.73 | 2 | 1691.782518 | 38.23 |
| IQETPIPTTFTTYNPNnDTTDTDGYGMNY-GAScAGVAASFSSHR | 1.24 | 3 | 4731.0853 | 23.42 |
| **Ps-Tox2** |  |  |  |  |
| ESLVELLDDFEGEDEWVTR | 5 | 2 | 2281.14409 | 73.95 |
| SYLYAQTCSNVESQK | 4.73 | 2 | 1777.88396 | 27.67 |
| YDAGTGEYHHATADVIQNPHFVPQQTPPQQTVNPAIR | 3.51 | 5 | 4100.13135 | 36.62 |
| WDYVEFWPCDIYNPYQK | 3.04 | 2 | 2323.11870 | 51.73 |
| FKDLPTIPSQLITTQGGYCLAPTQSR | 2.84 | 3 | 2893.59464 | 39.61 |
| SSGTNIVLDANK | 2.20 | 1 | 1218.68420 | 30.76 |
| FKDLPTIPSQLITTQGGYCLAPTQSR | 2.51 | 3 | 2894.54148 | 48.20 |
| **Ps-eTox1** |  |  |  |  |
| TIENFLPYPDYQINLIIELCQNILAR | 4.27 | 3 | 3165.75254 | 80.34 |
| AWHAGVSAFQDR | 2.32 | 2 | 1344.70976 | 32 |
| **Ps-eTox2** |  |  |  |  |
| SYLYAQTCSNVESQK | 3.03 | 2 | 1777.85198 | 28.18 |

^a^ SEQUEST correlation score.

^b^ Charge state values.

^c^ Precursor mass in Dalton.

^d^ Time of retention in minutes.
